# Soil Respiration and Organic Carbon Dynamics with Grassland Conversions to Woodlands in Temperate China

**DOI:** 10.1371/journal.pone.0071986

**Published:** 2013-08-23

**Authors:** Wei Wang, Wenjing Zeng, Weile Chen, Hui Zeng, Jingyun Fang

**Affiliations:** 1 Department of Ecology, College of Urban and Environmental Sciences, and Key Laboratory for Earth Surface Processes of the Ministry of Education, Peking University, Beijing, China; 2 Key Laboratory for Urban Habitat Environmental Science and Technology, Peking University Shenzhen Graduate School Shenzhen, China; Lakehead University, Canada

## Abstract

Soils are the largest terrestrial carbon store and soil respiration is the second-largest flux in ecosystem carbon cycling. Across China's temperate region, climatic changes and human activities have frequently caused the transformation of grasslands to woodlands. However, the effect of this transition on soil respiration and soil organic carbon (SOC) dynamics remains uncertain in this area. In this study, we measured *in situ* soil respiration and SOC storage over a two-year period (Jan. 2007–Dec. 2008) from five characteristic vegetation types in a forest-steppe ecotone of temperate China, including grassland (GR), shrubland (SH), as well as in evergreen coniferous (EC), deciduous coniferous (DC) and deciduous broadleaved forest (DB), to evaluate the changes of soil respiration and SOC storage with grassland conversions to diverse types of woodlands. Annual soil respiration increased by 3%, 6%, 14%, and 22% after the conversion from GR to EC, SH, DC, and DB, respectively. The variation in soil respiration among different vegetation types could be well explained by SOC and soil total nitrogen content. Despite higher soil respiration in woodlands, SOC storage and residence time increased in the upper 20 cm of soil. Our results suggest that the differences in soil environmental conditions, especially soil substrate availability, influenced the level of annual soil respiration produced by different vegetation types. Moreover, shifts from grassland to woody plant dominance resulted in increased SOC storage. Given the widespread increase in woody plant abundance caused by climate change and large-scale afforestation programs, the soils are expected to accumulate and store increased amounts of organic carbon in temperate areas of China.

## Introduction

Soils are the largest store of carbon in the biosphere [1], so small changes in soil organic carbon (SOC) storage will profoundly influence atmospheric CO_2_ concentrations and potentially influence the global climate [2]. Moreover, soil respiration is the second largest flux of carbon between terrestrial ecosystems and the atmosphere [3]. Global changes have substantially impacted soil respiration and, in turn, SOC dynamics [4], [5]. However, soils are the largest source of uncertainty in the terrestrial carbon balance [6].

Natural and anthropogenic-induced vegetation-type conversions are among the most important components of global changes [7]. The shifts between grasslands and plant communities dominated by woody vegetation are one of the most frequent occurring vegetation transition types [8], [9], [10], [11], [12]. For instance, deforestation is believed to be a major anthropogenic source of CO_2_ to the atmosphere [13], [14], [15], [16], [17]. In contrast, large scale forest expansion and re-growth may be important sources for the missing carbon sink [18], [19]. Vegetation-type conversions influence the balance of organic carbon in soil and hence may cause changes in soil respiration [20], [21]. Changes in vegetation-type are expected to have major effects on the terrestrial carbon balance [22].

Shifts in vegetation types may profoundly affect the dynamics of soil respiration and SOC by influencing soil microclimate and the production and transfer of aboveground photosynthate to belowground [23], [24], [25], [26], [27]. However, the direction of changes in the soil respiration and the consequent changes in organic carbon storage in soil within adjacent grass-woody vegetative transition is still controversial [28], [29], [30]. The inconsistencies may, to a large degree, be caused by the differences in the various locations and the types of transition occurring [31].

Because regional aspects of the global carbon cycle are drawing increasing scientific and political interest, there is a strong impetus to better understand how land use change effects China's carbon balance [32], [33], [34]. However, few reports on soil respiration and SOC dynamics are available. Furthermore, the currently available studies were mainly conducted in China's southern tropical and sub-tropical areas [35], [36], [37], [38], [39], [40]. Nevertheless, the temperate areas of northern China are also experiencing frequent, diverse and continuous transitions in the vegetation types, which should substantially affect SOC dynamics and soil respiration in this area. Since the 1970s, the Chinese government has implemented several ecological restoration projects, including the Three-North Shelterbelt Program covering 41% area of the country, across the temperate regions of China that receive less than 400 mm of precipitation annually. These reforestation and afforestation activities were believed to influence carbon cycling and carbon storage in this area [41], [42]. In addition, the study of dynamics of organic carbon in soil shows the level of organic carbon in soil is relatively sensitive to increasing temperatures in the temperate climatic zone [43]. Therefore, evaluating how large-scale transitions of vegetation types influence soil respiration and consequent SOC storage is critical to calculating temperate China's carbon budget under the scenario of global change.

In this study, we quantify soil respiration and SOC dynamics from five adjacent grass-woody vegetation types in the temperate areas of northern China. We aimed to 1) measure annual soil respiration as well as SOC storage and residence time, and 2) explore the major drivers for the variations in soil respiration among different vegetation types. We hypothesized that 1) soil respiration as well as SOC storage and residence time were higher in woody vegetation types than in grasslands, and 2) vegetation-mediated change in soil microenvironments was a major driver for the variation of soil respiration.

## Materials and Methods

### Ethics Statement

The administration of the Saihanba Forestry Center gave permission for this research at each study site. We confirm that the field studies did not involve endangered or protected species.

### Site description and land-use history

The study was conducted at the Saihanba Forestry Center in Hebei Province and Inner Mongolia Autonomous Area, northern China (117°12′–117°30′E, 42°10′–42°50′N, 1,400 m a.s.l.). The study area has a semi-arid and semi-humid temperate climate and lies in a typical forest-steppe ecotone on predominately sandy soils with long and cold winters (November to March), and short springs and summers. Annual mean air temperature and precipitation from 1964 to 2004 were −1.4°C and 450.1 mm, respectively.

**Figure 1:**
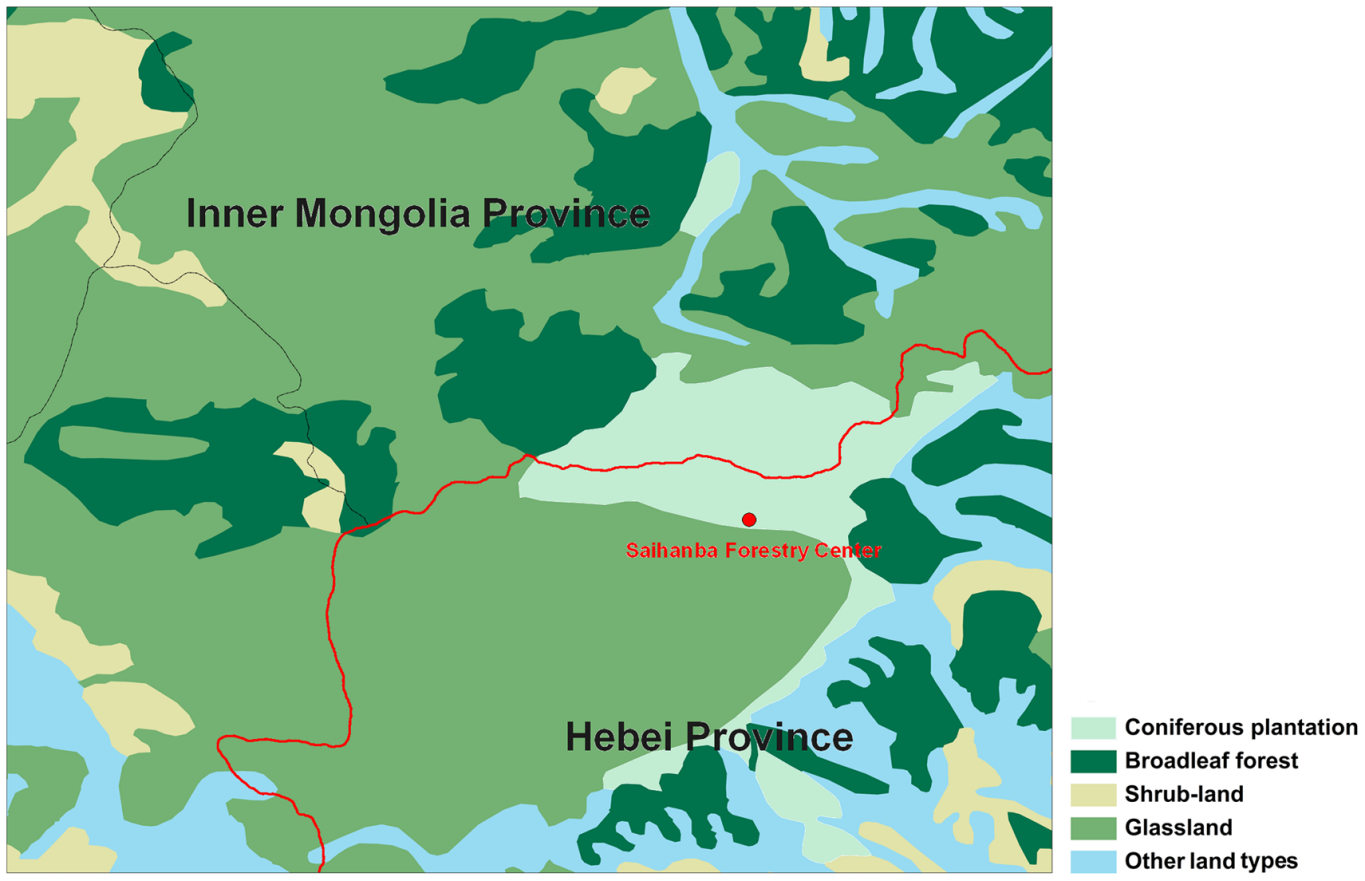
Current land-use patterns at the study site. From a 1∶1,000,000 scale map of the vegetation types of China [72].

This area contains the largest of plantation forests in China, with evergreen *Pinus sylvestris* L. var. *mongolica* Litv. (Mongolia pine) and deciduous *Larix principis-rupprechtii* Mayr (larch) as dominant species; secondary deciduous forests mainly consist of *Betula platyphylla* Sukaczev (birch). In addition, shrublands dominated by *Rosa bella* Rehd. et Wils. (Solitary rose) and *Malus baccata* (L.) Borkh. (Siberian crabapple) and meadow grasslands are also very common (Fig. 1). The herbaceous layers of Mongolia pine and larch are similar, and are composed of *Sanguisorba officinalis* L. (Radix Sanguisorbae), *Thalictrum aquilegifolium* L., *Agrimonia pilosa* Ledeb. and *Carex stenophylla* Wahlenb., while the herbaceous layer of birch is made up of *Agrimonia pilosa* Ledeb. and Radix Sanguisorbae. The herbaceous layer of Siberian crabapple is dominated by *Veronica linariifolia* Pall. ex Link, *Galium verum* L., *Heteropappus hispidus* (Thunb.) Less., *Trollius chinensis* Bunge, and *Bupleurum chinense* DC. The herbaceous layer of Solitary rose consists of *Leymus chinensis* (Trin.) Tzvelev. The meadow grassland is zonal vegetation dominated by *L. chinensis*.

The current land-cover pattern resulted from both natural and human-induced vegetation type transitions: from ∼5900 to ∼2900 ^14^C years BP, the original deciduous broadleaf forest (DB) were gradually replaced by evergreen coniferous forest (EC) and deciduous coniferous forest (DC) in those places when climate changed from humid to arid; after ∼2900 ^14^C years BP, EC and DC shifted to grassland (GR) in some drier places [44]. In the late 1900 s, the remaining primary forests were harvested by large scale industrial logging and initially became grasslands, but more recently the grasslands have been replaced by secondary SH, DB and plantations of EC and DC. Furthermore, based on the trends for increasing temperature and precipitation in this area [45], together with the large-scale reforestation and afforestation policy of the Chinese government [42], the cover area of woody vegetation types is predicted to increase in the future.

### Experimental plot design

**Figure 2:**
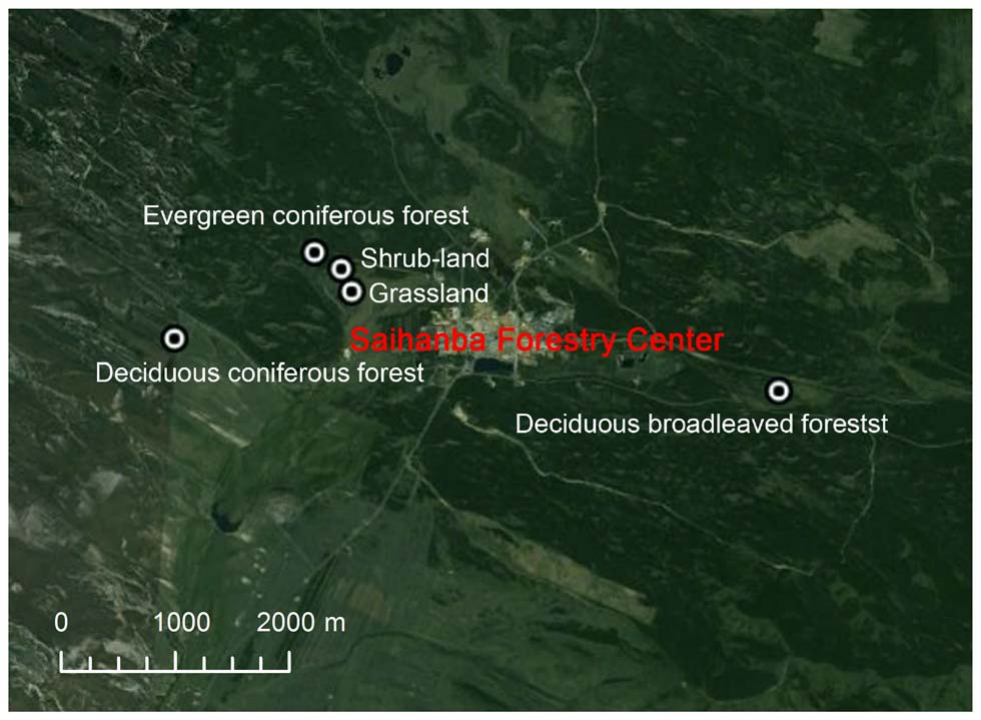
The location of five vegetation types in Saihanba Forestry Center.

The abundant vegetation types co-occurring in our study area provide an excellent opportunity to examine how ecological processes respond to changes in vegetation type. We selected five adjacent grass-woody vegetation types (Fig. 2) to study the influences of vegetation type transitions on biogeochemical processes. All sites for these vegetation types were less than 5 km apart to ensure each site had the same climatic and edaphic condition. Table 1 summarizes the characteristics and species composition of each vegetation type. Three replicates were designed for each of five vegetation types including: GR (*L. chinensis*), SH (*R. bella* & *M. baccata*), EC (∼15 year old *P. sylvestris* var. *mongolica*), DC (∼15 year old *L. principis-rupprechtii*), and DB (∼45 year old *B. platyphylla*). Each 20 m×20 m plot was sampled with five subsamples (i.e. soil respiration measurement collars).

**Table 1:** Site characteristics and physical and chemical properties of topsoil (0–20 cm).

Site	Vegetation types	Domain species	NDVI	ST (°C)	SWC (%)	SOC (g m^−2^)	STN (g m^−2^)	Soil pH	SBD* (g cm^−3^)
GR	grassland	*Leymus chinensis*	0.44^a^	3.8^a^	8.4^a^	1476.7^a^	136.5^a^	6.28^a^	0.92^a^
SH	shrubland	*Rosa bella Rehd.* et *Wils* & *Malus baccata* *	0.38^b^	3.4^a^	12.6^b^	1841.5^a^	189.4^b^	6.30^a^	0.71^b^
EC	∼15 yr old evergreen coniferous plantation	*Pinus sylvestris* var. *mongolica*	0.55^c^	3.2^a^	7.3^a^	2022.7^a^	146.2^a^	6.45^a^	0.98^a^
DC	∼15 yr old deciduous coniferous plantation	*Larix principis-* *rupprechtii*	0.36^b^	3.6^a^	7.8^a^	2993.9^b^	172.8^a^	6.30^a^	1.06^a^
DB	∼45 yr old deciduous broadleaved forest	*Betula* *platyphylla*	0.51^d^	1.7^b^	19.7^c^	2830.7^b^	287.7^c^	5.92^b^	0.69^b^

* SH contained two different dominant species in separate plots and the results of those plots were averaged in our study.

NDVI  =  Normalized Difference Vegetation Index, soil temperature  =  ST, SWC  =  soil water content, SOC  =  soil organic carbon, STN  =  soil total nitrogen, SBD  =  soil bulk density. Different lowercase letters indicated significant differences (*P*<0.05).

### Soil respiration, soil temperature and moisture

Soil respiration (SR) was measured using a Li-8100 soil CO_2_ flux system (LI-COR Inc. Lincoln, NE, USA) from Jan 2007 to Dec 2008. During the growing season (April to October), five polyvinyl chloride (PVC) collars (10 cm inside diameter, 6 cm height above the soil surface) were inserted 3 cm into the soil in each plot and were left in the same locations throughout the study period. These five PVC collars were placed in each plot, one in the center and one in each corner. Living plants inside the collars were clipped at the soil surface 1 day before each measurement to exclude the effect of aboveground vegetation. SR was measured every 10–15 days. Measurements were made between 08∶00 and 11∶00 am (based on our measurements of diurnal changes in SR, data not shown) to minimize the daily variation in SR and obtain mean daily SR. For each measurement, respiration rates were calculated as means of three plots for each stand. During winter (November to March), longer soil collars (determined by snow depth, less than 30 cm) were inserted into the soil surface and stabilized for 24 h before measurement of the winter SR [46], [47]. The Li-8100 soil CO_2_ flux system was kept in an isolated and heated container to keep its temperature above freezing point.

Soil temperature (ST) was recorded during respiration measurements near each collar at 5 cm soil depth with the LI-COR 8100 temperature probe. Continuous measurements of ST at 5 cm depth were recorded at 30-min intervals with StowAway loggers (Onset Comp. Corp., Bourne, MA, USA) inserted in the soil near one collar at each study site. Soil volumetric water content (SWC) at a depth of 0–10 cm was measured using time domain reflectometry (Soil Moisture Equipment Corp., Santa Barbara, CA, USA). SWC was only obtained during the growing season because the probe could not be fully inserted into the frozen soil in winter.

### Soil sampling and measurements

Because the carbon stored in topsoil is the carbon pool that is most sensitive to land management practices [48], [49], we sampled mineral soils at depths of 0–10 and 10–20 cm from five random locations per plot using 5.8-cm diameter soil cores during the summers of 2007 and 2008. Soil bulk density (SBD) of the two soil horizons was quantified in all soil surveys from the mass of the oven-dry soil (105°C) divided by the volume of the soil cores. Next, all plant materials were removed from fresh soil samples, and soil was passed through a 2-mm sieve. *In situ* root biomass per unit area was determined by the entire root biomass in the soil core divided by the cross section area of the core. Soil pH was determined from air-dried soil samples in distilled H_2_O solution, with a pH meter (Model PHS-2, INESA Instrument, Shanghai, China). The SOC content and soil total nitrogen (STN) were determined from oven-dried soil samples with an elemental analyzer (Vario EL III Universal CHNOS Elemental Analyzer, Elementar, Hanau, Germany). The mass-based SOC and STN were converted into area-based with soil bulk density of each horizon (0–10 and 10–20 cm depth).

NDVI (Normalized Difference Vegetation Index) data

NDVI is derived from the red: near-infrared reflectance ratio: 
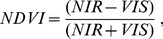
(1)where NIR and VIS stand for the spectral reflectance measurements acquired in the near-infrared and visible (red) regions of the spectrum, respectively [50]. The NDVI depends on photosynthesis and is closely correlated to GPP [51], [52]. We calculated the mean annual NDVI of each vegetation type from 2007–2008 to analyze the influence of aboveground carbon input on SR. Because the five vegetation types studied here covered large areas and had uniform distributions and sparse understories, NDVI of 16-Day L3Global 250 m product (MOD13 Q1) could well represent our plot measurements. We downloaded satellite data from our study period from https://wist.echo.nasa.gov/api. Harmonic (Fourier) analysis was used to remove the considerable noise remaining in the NDVI time series from satellite data to obtain reasonably smooth continuous data [53].

### Statistical analysis

We examined the relationships between SR and ST by fitting exponential functions to the data from each vegetation type using the following equation: 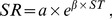
(2)where SR is observed soil respiration (plot-wide averages measured periodically throughout the year), ST is the concurrently measured soil temperature (5 cm depth), with a and β being the fitted parameters obtained using least squares nonlinear regression with SigmaPlot V. 8.02.

Annual SR was estimated with the yearly period continuously measured ST and the exponential function between SR and ST for each vegetation type. The mean residence time of SOC was estimated for each vegetation type by dividing the mass of SOC in the top 20 cm of the soil profile by the heterotrophic respiration flux, which equals the total SR minus root respiration. Root respiration of each vegetation type was obtained based on the estimates from Wang *et al.* [54], who measured excised root respiration for all the five vegetation types we studied at the same sites during the same period.

To analyze the environmental differences among vegetation types, principal component analysis (PCA) was performed on correlations among ST, SWC, NDVI, SOC, STN, soil pH and SBD, and the five vegetation types were ordered by their scores on the first two principal components. The relationships between annual SR and ST, SWC, NDVI, SOC, STN and live fine root biomass were examined by linear regression. The differences of SR among vegetation types were tested using one-way ANOVA. We used the averaged value of the five subsamples in each plot to conduct statistical analysis. All statistical analyses were performed with a significance level of 0.05, using SPSS software (2009, ver. 18.0, SPSS Inc., Chicago, IL, USA).

## Results

### Microenvironment of different vegetation types

The annual average ST at 5 cm deep was not significantly different between grassland and woody vegetation types, with one exception (Table 1). The DB habitat had significantly lower ST at 5 cm deep and higher SWC at 10 cm deep. No significant differences in SWC occurred among GR, EC and DC habitats. The SH and DB habitats had lower soil bulk density than other vegetation types, but the DC forest and DB forest showed higher SOC content (Table 1, *P*<0.05). Furthermore, STN content was highest in DB forest, and its pattern was consistent with that of SWC across different vegetation types. There is no significant difference in soil pH among all vegetation types (Table 1).

**Figure 3:**
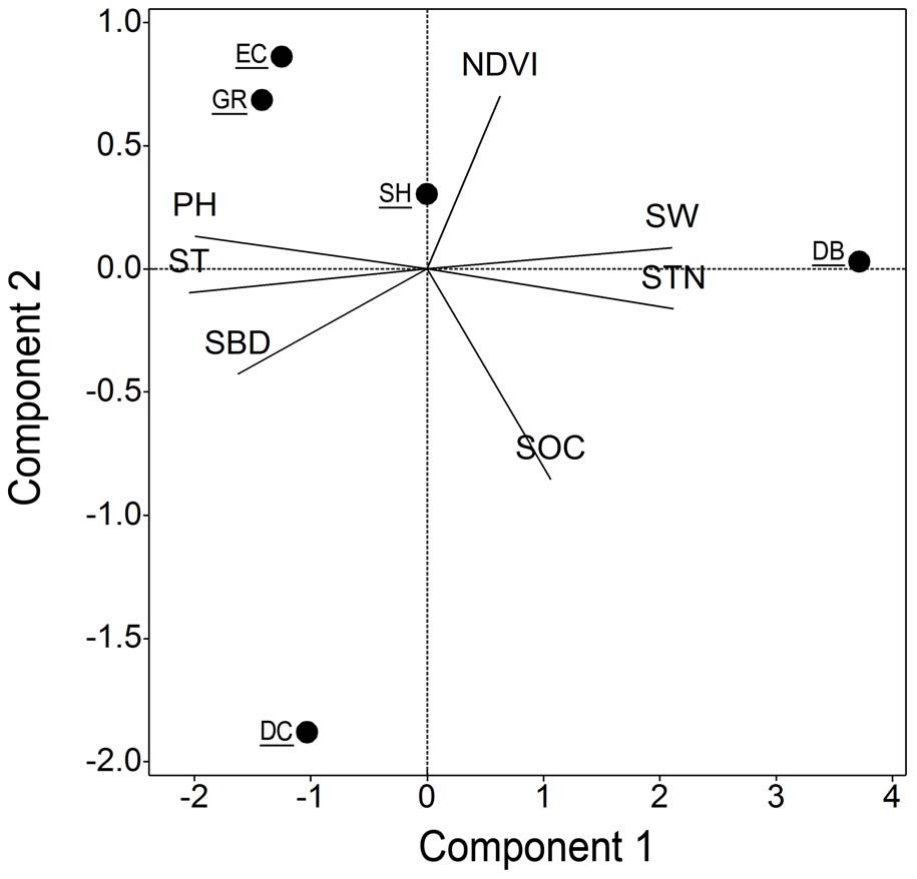
Principal component analysis of site properties. Score plot of five vegetation types during Principal Component Analysis of site properties, including normalized difference vegetation index (NDVI), soil temperature (ST), soil water content (SWC), soil organic carbon (SOC), soil total nitrogen (STN), soil pH, and soil bulk density (SBD). Habitats were grassland (GR), shrubland (SH), evergreen coniferous forest (EC), deciduous coniferous forest (DC), and deciduous broadleaved forest (DB).Seasonal dynamics of soil respiration

PCA identified three significant principle components (eigenvalue >1) of variations (Fig. 3). The first two principle components explained 83% of the total variance in the dataset (Table 2, Table 3). The first principal component was mainly associated with the differences in ST, SWC, STN and soil pH across different vegetation types. The second and third principal components were correlated to SOC and NDVI, respectively. Among the four woody vegetation types, EC is the most similar environmentally to GR, followed by SH. However, DB and DC were the different from GR along the first and second principal component, respectively.

**Table 2:** The proportion of variation explained from principal component analysis on the seven environmental variables.

Component	Eigenvalue	Proportion	Cumulative
1	4.61	0.66	0.66
2	1.21	0.17	0.83
3	1.01	0.15	0.98
4	0.16	0.02	1.00

**Table 3:** The loading scores of traits on each component from principal component analysis on the seven environmental variables.

Variable	Component 1	Component 2	Component 3
NDVI	0.14	0.58	−0.71
ST	−0.44	−0.08	0.28
SWC	0.46	0.07	0.18
SOC	0.23	−0.71	−0.37
STN	0.46	−0.14	0.02
pH	−0.43	0.11	−0.10
SBD	−0.35	−0.35	−0.49

NDVI  =  normalized difference vegetation index, ST  =  soil temperature, SWC  =  soil water content, SOC  =  soil organic carbon, STN  =  soil total nitrogen, pH  =  soil pH, SBD  =  soil bulk density.

**Figure 4:**
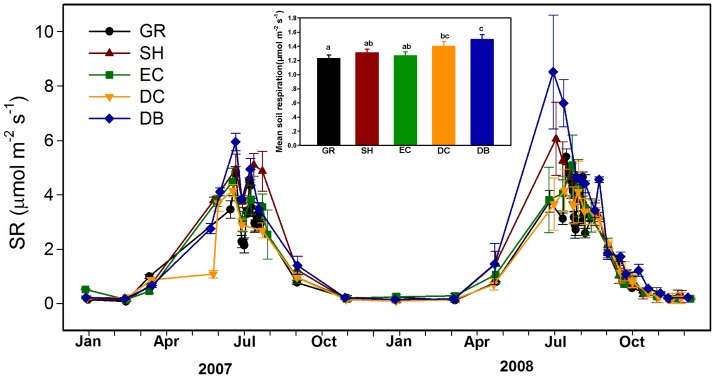
Seasonal dynamics of soil respiration (SR) among five adjacent vegetation types. Habitats are as listed in Fig. 2.

**Figure 5:**
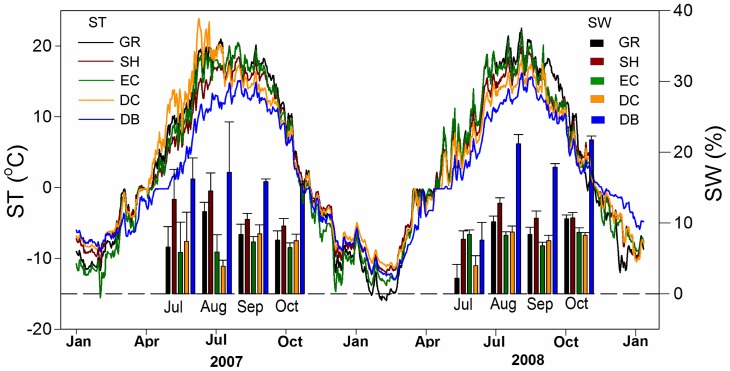
Seasonal dynamics of soil temperature and soil water content across five adjacent vegetation types. ST  =  soil temperature at 5 cm depth, SWC  =  soil water content at 10 cm depth. Habitats are as listed in Fig. 2.

**Figure 6:**
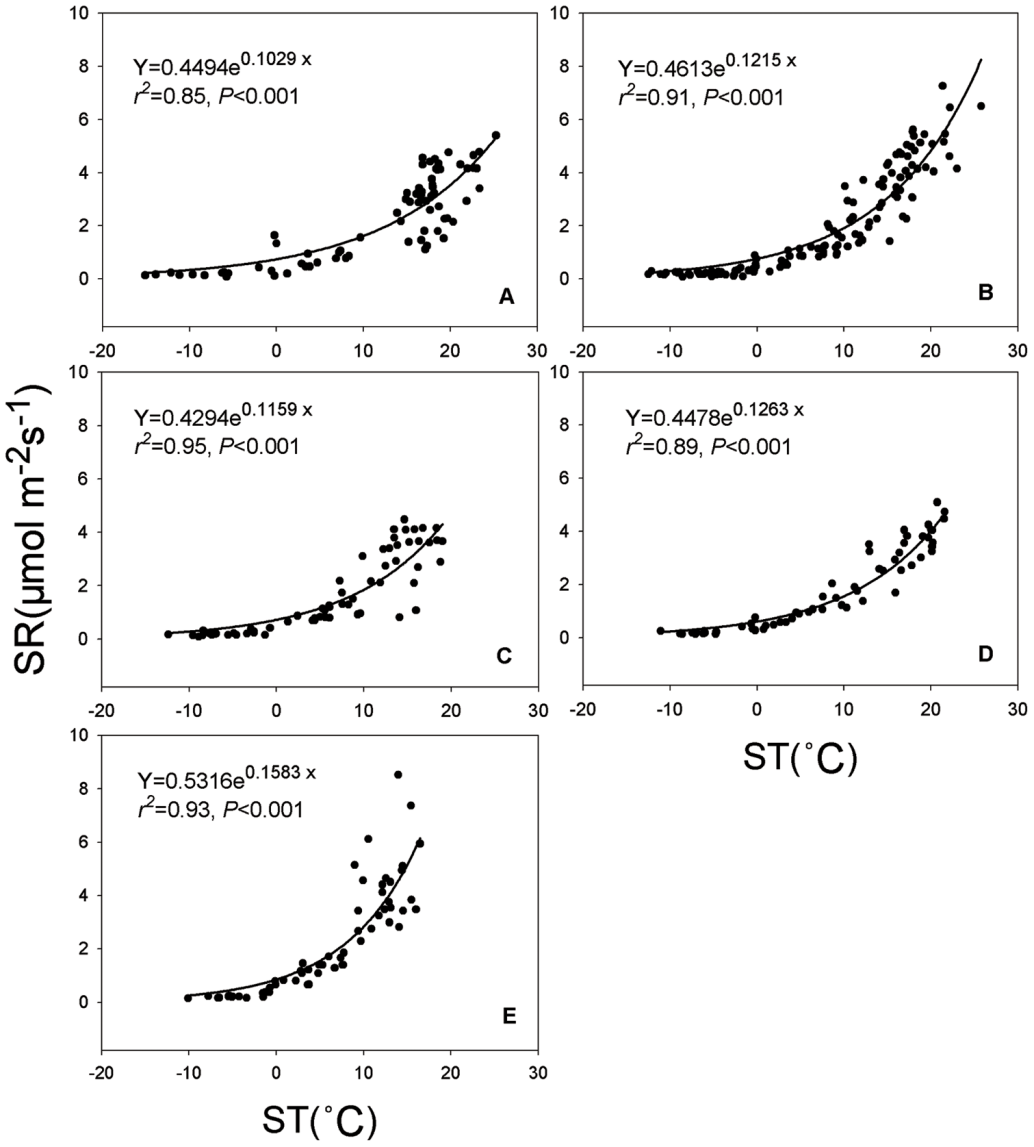
Relationships between the seasonal dynamics of soil respiration and soil temperature at 5 cm depth across habitats as listed in Fig. 2

As expected, SR (Fig. 4) and ST (Fig. 5) were higher in summer and lower in winter (Fig. 4). The mean SR showed significant differences among five vegetation types, in the order of GR < EC < SH < DC < DB (Fig. 4). The seasonal dynamics of SR were exponentially related to ST across different vegetation types, which explained 85%, 91%, 95%, 89% and 93% of the variation in SR for GR, SH, EC, DC and DB, respectively (Fig. 6).

### Annual soil respiration and soil organic carbon turnover

**Figure 7:**
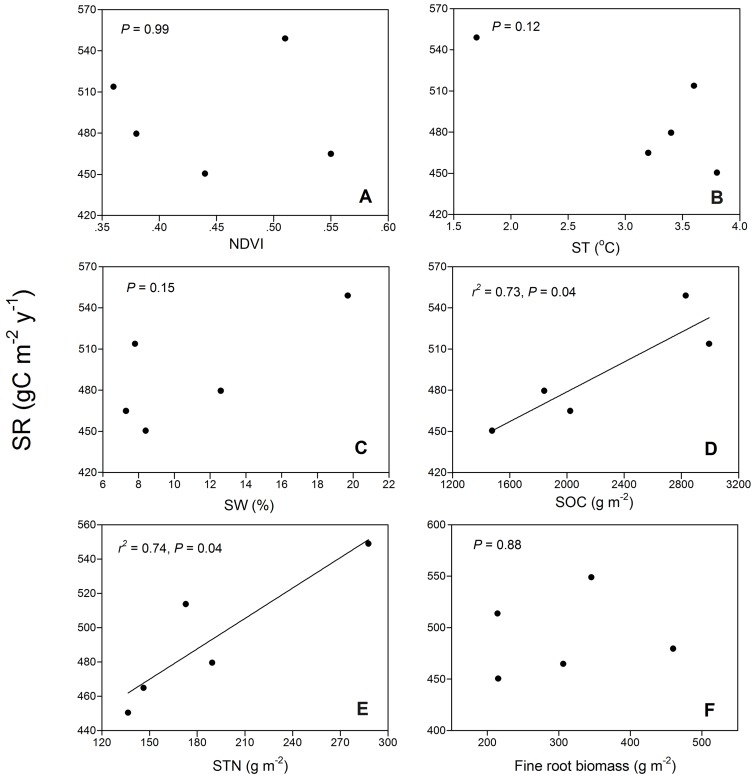
Relationships between annual soil respiration and NDVI (A), soil temperature at 5 cm depth (ST) (B), soil water content at 10 cm depth (SWC) (C), soil organic carbon (SOC) (D), soil total nitrogen (STN) (E) from the top 20 cm depth and fine root biomass at 0–30 cm depth (F) across the five adjacent vegetation types.

Annual SR was lower in GR than in the woody vegetation types (Table 4). The annual SR increased by 3% following the conversion from GR to EC, 6% to SH, 14% to DC, and 22% to DB. The variations in the annual SR among the five vegetation types were significantly correlated with SOC and STN, but not with ST, SWC, NDVI and fine root biomass (Fig. 7).

**Table 4:** Annual soil respiration, contribution of root respiration to total soil respiration, and soil organic carbon residence time in the top 20 cm of soil.

Site	SR (g C m^−2^ yr^−1^)	Root respiration contribution* (%)	HR (g C m^−2^ yr^−1^)	SOC (g m^−2^)	Residence time (yr)
GR	450.4^a^	4.7	429.0	1476.7^a^	3.4
SH	479.5^b^	23.5	366.8	1841.5^a^	5.0
EC	464.8^c^	18.3	380.0	2022.7^a^	5.3
DC	513.7^d^	24.0	390.2	2993.9^b^	7.7
DB	548.9^e^	17.9	450.5	2830.7^b^	6.3

* The estimates were derived from those of Wang *et al*. [54].

SR  =  annual soil respiration, HR  =  annual heterotrophic respiration, SOC  =  soil organic carbon, GR  =  grassland, SH  =  shrubland, EC  =  evergreen coniferous forest, DC  =  deciduous coniferous forest, DB  =  deciduous broadleaved forest. Different lowercase letters in mean SR and SOC indicated significant differences (*P*<0.05).

In contrast with the pattern of total SR, GR had higher annual heterotrophic respiration than SH, EC and DC, but lower than DB (Table 4). Because GR had a lower SOC content and higher heterotrophic respiration, the residence time of SOC in GR is shorter (3.4 yr), in comparison with the relatively longer time in woody vegetation types (5.0 yr in SH, 5.3 yr in EC, 6.3 yr in DB and 7.7 yr in DC, Table 4).

## Discussion

### Effects of vegetation types on soil respiration

Our estimates of annual SR ranged from 450.4 to 548.9 g C m^−2^ yr^−1^(Table 4), which fell into the range reported in temperate areas [55], [56]. Among the five vegetation types, GRs showed lower SR than woodlands, which contradicted with the conclusions made using a synthesis of global data that reported SR from various types of GRs was averaged about 20% greater than various types of forests [57]. Additionally, in a juniper woodland-grassland pair in Kansas (USA), SR from GRs was 38% higher than from woodlands [58]. Our findings therefore did not support earlier generalizations that state GRs tended to allocate large proportions of their photosynthate belowground, and this results in higher SR than occurs woodlands [58]. However, in a subalpine Australian ecosystem rates of respiration in woodland soils were twice more than those in nearby grassland soils, which is similar to our results [27].

**Figure 8:**
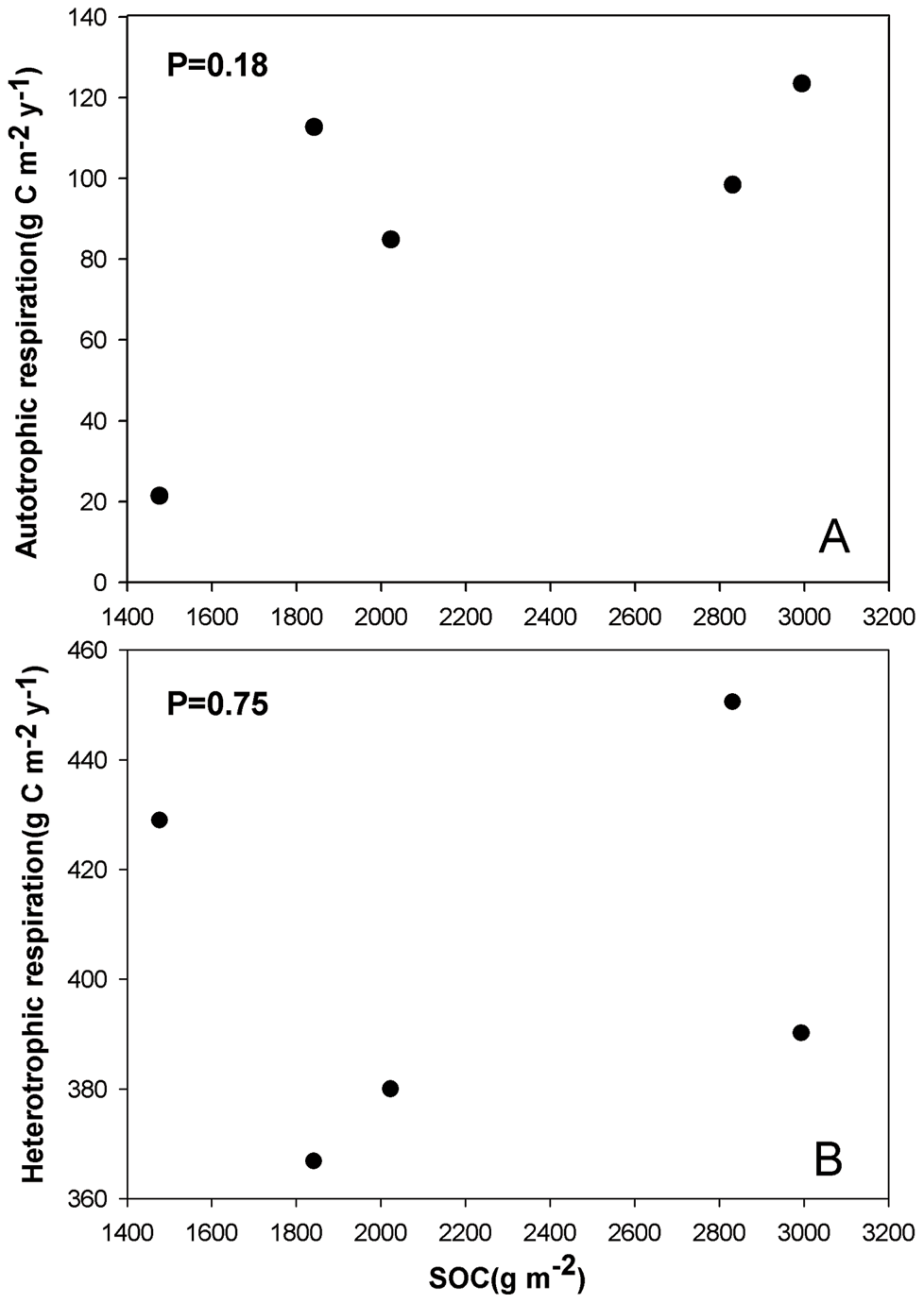
Relationships between heterotrophic respiration (A), autotrophic respiration (B) and soil organic carbon (SOC) across the five adjacent vegetation types.

Three factors, microclimate (ST and moisture), aboveground photosynthetic supply to roots and substrate availability have been known to be important controls on SR [57]. Our results suggested that SOC and STN were major contributing factors for the variations of SR among different vegetation types (Fig. 7). Heterotrophic respiration was the dominant component of total SR, ranging from 76% to 95% (Table 4). Therefore, we expected the correlation between SR and SOC was derived from the component of heterotrophic respiration, because heterotrophic respiration is a result of the mineralization of SOC that is stored in large stocks [59], [60], [61] while autotrophic respiration depends on fresh photosynthates [62]. However, we did not observe a correlation between HR and SOC (Fig. 8A). Moreover, we observed an increasing trend of root respiration with an increase in SOC (Fig. 8B) although statistical test was not significant. Therefore, root respiration may be the main driver of the differences in SR between the different vegetation types. The correlation between root respiration and SOC may be attributable to the fact that higher root respiration is connected to higher photosynthetic activity [63], [64], [65], which will increase the carbon input to soil and therefore SOC content. Therefore, accurate discrimination of root respiration and heterotrophic respiration from the total SR was critical for gaining an improved understanding of the driving factors of SR among different vegetation types. Our results thus indicate that when modeling SR across a temperate heterogeneous landscape, we should pay more attention to the differences in heterotrophic respiration and root respiration components among different vegetation types.

### Effects of vegetation types on soil carbon storage and turnover

Reported changes of SOC storage varied widely with reported increases [28], [30], [66], no change [58] and decreases [67], [68], [69] after grasslands were converted to woodlands. Our results showed that SOC storage was 92% higher in DB and 103% higher in DC compared with GR (Table 4), suggesting possible larger amounts of organic carbon are input to soil through litterfall and root turnover in woodlands than grassland. Moreover, grasslands have been reported to have stronger wind erosion than woodlands in our study area, which reduced the soil clay and silt content, possibly explaining the potential reductions of surface organic matter in our study area [70], [71]. However, no significant increase was observed when EC and SH (Table 4) were compared with GR, possibly because of the small sample size and large spatial heterogeneity seen in SOC in this study.

In our study area, mean residence time of the near-surface SOC pool in woody communities exceeded that of GR (Table 4). Similarly, McCulley *et al*. [66] also observed that both SR and mean residence time of the near-surface SOC pool in wooded communities (11 years) exceeded that of GRs (6 years) in a subtropical ecosystem. However, in paired juniper woodland and C_4_-dominated grassland sites, longer woodland topsoil residence time (33 years) was observed than in GRs (18 years) [58]. In addition, our estimates of SOC residence time were shorter than those of McCulley *et al*. [66] and Smith & Johnson [58], partly because of the differences in the estimates of heterotrophic respiration. In our study, heterotrophic respiration comprised a large portion of total SR (76% to 95%, Table 4), whereas, Smith & Johnson [58] assumed root respiration is 50% of SR and McCulley *et al*. [66] used three scenarios in which they assumed root respiration comprised 30%, 50%, or 70% of total SR.

## Conclusion

In conclusion, by determining SR and SOC dynamics in five adjacent vegetation types (GR, SH, EC, DC and DB) in the temperate area of northern China, we identified an increase in both annual SR and residence time of SOC from grassland to woody vegetation types. The increase in annual SR was coupled with changes in soil substrate availability (SOC and STN). The increases in SR suggest an increase in landscape-scale carbon emissions occurred during both natural and anthropogenic transitions occurred from grassland to plant communities dominated by woody vegetation. However, the SOC pool storage and its residence time also increased, suggesting a larger increase in carbon input than in carbon loss from the surface soil layer, thus implying an accumulation of SOC during grassland conversion into woodlands in temperate China.
