# Correction: Soil Respiration and Organic Carbon Dynamics with Grassland Conversions to Woodlands in Temperate China

**DOI:** 10.1371/annotation/d1c26c59-62e8-4c8d-96f2-439ca922b14a

**Published:** 2013-09-06

**Authors:** Wei Wang, Wenjing Zeng, Weile Chen, Hui Zeng, Jingyun Fang

There was an error in the Funding statement. The correct version of the Funding statement is available below.

This research was supported by the National Basic Research Program of China (No. 2013CB956303 and 2010CB950600), projects of the National Natural Science Foundation of China (31222011, 31270363 and 31070428), and projects supported by the Foundation for Innovative Research Groups of the National Natural Science Foundation of China (No. 31021001). The funders had no role in study design, data collection and analysis, decision to publish, or preparation of the manuscript. 

